# Human T-Lymphotropic Virus Screening of Blood Donations in England Between 2002 and 2021—Comparison of Screening Strategies

**DOI:** 10.1093/cid/ciaf053

**Published:** 2025-02-03

**Authors:** Heli Harvala, Katy Davison, Mhairi Webster, Claire Reynolds, Graham P Taylor

**Affiliations:** Microbiology Services, NHS Blood and Transplant (NHSBT), London, United Kingdom; Radcliffe Department of Medicine, University of Oxford, Oxford, United Kingdom; Infection and Immunity Unit, Institute of Biomedicine, Faculty of Medicine, University of Turku, Turku, Finland; NHSBT and UK Health Security Agency (UKHSA) Epidemiology Unit, UKHSA, London, United Kingdom; Microbiology Services, NHS Blood and Transplant (NHSBT), London, United Kingdom; NHSBT and UKHSA Epidemiology Unit, NHSBT, London, United Kingdom; Section of Virology, Department of Infectious Disease, Faculty of Medicine, Imperial College London, London, United Kingdom; National Centre for Human Retrovirology, Imperial College Healthcare NHS Trust, St Mary's Hospital, London, United Kingdom

**Keywords:** HTLV, blood donation screening in England, assay specificity, pooled testing

## Abstract

**Background:**

Human T-lymphotropic virus (HTLV) is associated with adult T-cell leukemia/lymphoma and myelopathy. Here we present virological and epidemiological data on HTLV screening of blood donations in England between 2002 and 2021, implemented to prevent its transmission via blood transfusion.

**Methods:**

Data on HTLV testing of blood donations was reviewed; it was initially conducted in pools (2002–2012) and subsequently using individual samples (all donors, 2013–2016; first-time donors and non-leucodepleted component donors, 2017–2021). Data included annual number of donations screened, initial and repeat reactives as well as confirmed positives. Further information, such as likely source of infection, was obtained for HTLV-positives.

**Results:**

Over the 20-year study period, a total of 30 679 741 blood donations were screened for HTLV in England. Under pooled screening strategy, the annual rate of repeat reactive donations remained <5:100 000. However, this rate increased to 51:100 000 with individual screening and further to 123:100 000 with selective screening. A total of 5032 samples were repeat reactive, of which 278 were confirmed HTLV-positives. Although the specificity under each scenario exceeded 99.9%, the rate of repeat reactives was around 50-fold higher in individual compared to pooled screening. Most HTLV infected were UK-born, most likely acquired their infection unknowingly through breast feeding or heterosexual intercourse with an individual associated with an HTLV-endemic country.

**Conclusions:**

These data highlight that pooled testing can be advantageous in low-prevalence settings due to its high specificity and reduced non-specific reactivity. Whether pooling is an applicable strategy to tackle the burden of HTLV infection in resource-poor, HTLV-endemic countries requires further investigations.

## Teaser

Screening over 30 million blood donations for human T-lymphotropic virus in England, 2002–2021, demonstrates the remarkable assay specificity of 99.985%. Pooled testing shows further advantages in low-prevalence settings due to its even higher specificity and reduced non-specific reactivity, making it more cost-effective.

Human T-lymphotropic virus types 1 and 2 (HTLV-1 and HTLV-2) are closely related enveloped retroviruses. Most HTLV infections are asymptomatic, and those infected are frequently unaware of their infection. However, HTLV-1 has been linked to disease through three major pathways: inflammation, proliferation and coinfection. Most etiological studies have been conducted in endemic countries where 0.25% and 3.8% of HTLV-1 infected people will develop HTLV-1-associated myelopathy/tropical spastic paraparesis (HAM/TSP) [1, 2], the best-known consequence of inflammation associated with HTLV-1 infection, but noting multiple other systems are also often affected. The life-time risk of developing adult T-cell leukemia/lymphoma is around 2.5% to 5% (ATLL) [3, 4]. HTLV-1 coinfection adversely impacts a range of bacterial, viral, helminthic and parasitic infections (reviewed by Rosadas and Taylor 2022 [5]).

HTLV-1 is endemic in many tropical and subtropical regions, including the Caribbean, Iran, Australia, Melanesia, South America, southern India, southern Japan, and Western Africa, with a seroprevalence often exceeding 10% [6]. HTLV-2 is more commonly found among indigenous American populations and has also spread among people who inject drugs. In endemic countries, HTLV-1 is predominantly acquired through vertical transmission, especially via breastfeeding, or through unprotected sexual contact. Although HTLV-1 infection is considered rare in much of Europe, it is endemic in Romania [7]. Elsewhere in Europe, most HTLV-1 infections are identified in persons migrating from endemic areas or has been acquired via their sexual partners linked to endemic areas. For example, over 80% of HTLV-positive individuals identified via blood donor or antenatal screening were from or had sexual partners from the HTLV-1 endemic areas [8, 9]. Furthermore, HTLV can also be transmitted through blood transfusion, organ, stem cells or bone marrow transplantation as well as cells of reproductive origin.

HTLV infection is usually diagnosed through the detection of HTLV-specific antibodies. HTLV-1 testing was introduced into clinical practice in the United Kingdom in 1986 [10], whereas routine blood donation testing for HTLV antibodies began in 2002 [11]. Despite that, most HTLV infections remain undiagnosed in the United Kingdom. This is based on a previous estimate that up to 30 000 people are living with HTLV in the United Kingdom [12] but < 100 HTLV diagnoses are made each year in the United Kingdom [13, 14]. Although the sensitivity of serological HTLV assays approaches 100%, the reported specificity of antibody assays remains just below 100%. This can be problematic in a low-prevalence population such as the United Kingdom, and for these reasons, it is important to apply more than 1 assay to confirm HTLV-1 (and HTLV-2) positivity. In UK practice, HTLV diagnosis is usually confirmed using 2 different immunoassays, followed by a western blot and/or polymerase chain reaction (PCR) to detect proviral DNA in a cellular sample [13, 15].

This article focuses on HTLV screening of blood donations by NHS Blood and Transplant (NHSBT) in the England, implemented in 2002. Initially screening was performed on pooled plasma samples from 48 donations prepared for viral molecular screening. In 2009, plasma pool size utilised for HTLV screening was reduced to 24 donations to reflect a change in the molecular screening system requiring smaller pool size than previously used. In 2013, HTLV screening moved from pooled to individual testing utilizing fully automated serological platforms for the first time. The HTLV screening strategy was reviewed two years later. An extremely low HTLV seroprevalence, rarity of seroconversions and lack of documented transfusion transmitted HTLV infection via leucodepleted components in the England prompted a change from universal screening to testing donations from first-time donors and donations made into components that could not be leucodepleted only from 2017 [16].

Here we determine the specificity of the algorithms used for anti-HTLV antibody screening of blood donations in the England between 2002 and 2021. We also review the epidemiology of the blood donors confirmed to be HTLV-positive and report the likely sources of HTLV infection in a low-endemic country.

## METHODS

Blood donation screening strategies applied by NHSBT regional laboratories in the England between 2002 and 2021 were reviewed. In brief, HTLV antibody screening was performed using the Abbott Murex HTLV-1/HTLV-2 enzymatic immunoassay (2002–2013), and the Abbott Prism HTLV-1/HTLV-2 chemiluminescent immunoassay (2013–2021). Testing was initially conducted in pools of 48 (2002–2008) or 24 plasma samples (2009–2012) and subsequently using individual samples (all donors, 2013–2016; first-time donors and components, which inherently cannot be leucodepleted, namely, granulocytes, 2017–2021). Reactive pools were resolved into an individual positive sample. Samples screened individually and found reactive in a screening assay were retested in duplicate and repeat reactive samples subjected to confirmatory testing at the NHSBT Microbiology Services Laboratory in London. Confirmatory testing included 2 further immunoassays (Murex HTLV-1/HTLV-2 by Diasorin and HTLV-1/HTLV-2 Ab version Ultra by Diapro), Western Blot (MP Diagnostics) and in-house PCR for proviral DNA using whole blood when required following the confirmatory testing algorithm. Samples were reported as confirmed positive when all confirmatory assays were positive.

All confirmed HTLV-positive blood donors were invited for a post-test discussion, usually by telephone, with the NHSBT Microbiology Services clinical team. The aim of this discussion was to notify donors, identify the most likely source of their infection, provide information about HTLV and offer an opportunity to be reviewed at one of the regional HTLV clinics established by the Department of Health, coordinated by the National Centre for Human Retrovirology, Imperial College Healthcare NHS Trust, London (https://www.imperial.nhs.uk/sexual-health/sexual-health/htlv).

Data on blood donations screened in England, 2002–2021, were obtained from the joint NHSBT/UKHSA Epidemiology Unit through the national surveillance scheme. This included information about donations tested and found positive for HTLV along with characteristics of confirmed positive donors and the probable source of their infection provided by the donor at the post-test discussion [17]. Data reviewed in this study included the total number of blood donations screened every year, the number of initial and repeat reactive donations as well as confirmed positives. Further information on HTLV-positive donors including gender, age group, country of birth, ethnicity, and likely source of their infection was also obtained.

This analysis was undertaken for clinical audit purposes to assess and improve service under permissions granted in paragraph 7 of Chapter 2 to the Data Protection Act 2018. According to that, NHSBT, as a government body, may process personal data as necessary for the effective performance of a task carried out in the public interest. As part of the written consent for donation, donors agree to using their data for additional purposes including clinical audit to assess and improve services. This analysis was not considered research by the NHS.

## RESULTS

**Figure 1.**
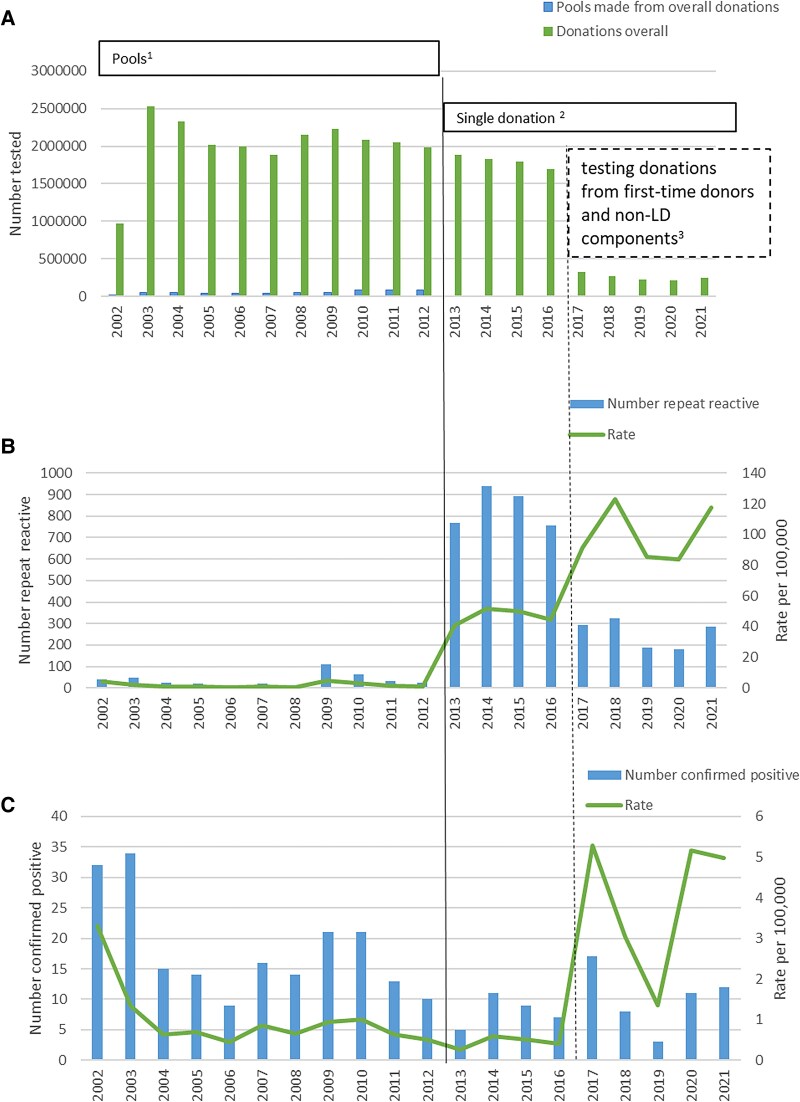
HTLV screening of blood donations (*A*), number and rate of repeat HTLV-reactive donations (*B*), and number of confirmed HTLV-positive donors (*C*) identified in England by year, 2002–2021. *A*, A number of blood donations (gray bars) and pools (black bars) tested for anti-HTLV by year of testing, England 2002 to 2022 (N = 30 679 741). *B*, The number (black bars) and rate per 100 000 blood donations (black line) found to be repeat reactive for anti-HTLV by year of testing, England 2002 to 2022 (n = 5032). *C*, The number (black bar) and rate per 100 000 blood donations (black line) confirmed positive for anti-HTLV by year of testing, England 2002 to 2022 (n = 278). All samples were resolved either to positives, or negatives. Footnotes for the figure. ^1^All donations—screened in minipools Aug 2002–Feb 2013. ^2^All donations—screened individually March 2013–Dec 2016. ^3^First-time donor donations and those for non-leucodepleted—screened individually Jan 2017–Dec 2021. Abbreviation: HTLV, human T-lymphotropic virus.

Over the 20-year study period, a total of 30 679 741 blood donations were screened for anti-HTLV antibodies in England. In the first full year of testing in 2003, samples of 2.5 million donations were made into 54 279 pools for screening (Figure 1). In the years after, around 2 million donations and 45 000 pools were tested annually. From 2009, the pool size was reduced from 48 to 24 donations, which increased the number of pools tested. In 2013 pooling was discontinued, and about 1.8 million individual donations were screened each year until 2017 when only donations from first-time donors and those intended for components which could not be leucodepleted were tested, totalling approximately 30 000 donations each year. Under pooled screening strategy, the rate of repeat reactive donations remained consistently low at fewer than 5 per 100 000 donations per year. However, with the change to single-donation screening, this rate increased to 51 per 100 000 for all donations in 2014 and further to 123 per 100 000 for donations from first-time and non-leucodepleted component donors in 2018.

Between 2002 and 2021, a total of 5032 samples were repeat reactive, of which 278 were confirmed HTLV-positives (Table 1). The remaining 4754 samples had non-specific reactivity (94.48%), and the overall specificity of the screening assay was estimated at 99.985%. Although the specificity under each scenario exceeded 99.9%, the rate of repeat reactives was around 50-fold higher in individual compared to pooled screening.

**Table 1.** Human T-lymphotropic Virus (HTLV) Screening of Blood Donations, Number and Rate of Repeat HTLV-reactive Donations and Number of Confirmed HTLV-Positive Donations Identified in England by Testing Protocol and Specificity, 2002–2021 (N = 30 697 741)

UK Blood Donations Screening Protocol	All Donations—Screened in Minipools	All Donations—Screened Individually	^a^Selected Donations—Screened Individually	All Together
**Dates of testing**	**Aug 2002–Feb 2013**	**March 2013–Dec 2016**	**Jan 2017–Dec 2021**	**Aug 2002–Dec 2021**
**Number of pools tested**	**624 445**	**0**	**0**	**624 445**
**Number of donations tested**	**22 511 003**	**6 908 058**	**1 260 680**	**30 679 741**
**Number of initial reactive donations**	**623**	**7945**	**2603**	**11 171**
**Rate per 100 000**	2.77	115.01	206.48	36.41
**Number of repeat reactive donations**	**413**	**3349**	**1270**	**5032**
**Rate per 100 000**	1.83	48.48	100.74	16.40
**Number of confirmed positive donations**	**197**	**30**	**51**	**278**
**Rate per 100 000**	0.88	0.43	4.05	0.91
**Number of non-specific reactives**	**216**	**3319**	**1219**	**4754**
**Non-specific reactives from repeat reactives (%)**	52.30	99.10	95.98	94.48
**Specificity (%)**	**99.999**	**99.952**	**99.903**	**99.985**

^a^Selected screening: donations from first-time donors and those used for non-leucodepleted components.

The 278 anti-HTLV-positive donations reported from the Microbiology Services laboratory corresponds to a rate of 0.91 per 100 000 donations tested (Table 1). Among these, 274 were included in the positive blood donor surveillance for England; 4 were excluded as 2 were donors living in Wales, and 2 were from donors who had given plasma for medicine. The highest rates of confirmed cases were for 2017 to 2021 when screening first-time and non-leucodepleted component donors, 4.05 per 100 000 donations tested.

Most positive donors were infected with HTLV-1 (247/274, 90%), were female (195/274, 71%), UK-born (138/274, 51%) and with a mean age of 43 years (range: 17–70 years). Almost all confirmed HTLV-positives were first-time and previously HTLV-untested donors (262/274). New diagnosis of HTLV infection was documented in 11 repeat donors: a seroconversion within a year from the previous donation was confirmed for 5, seroconversion within 2–3 years of previous donation for 2, and seroconversion over 3 years ago for 2 donors. Two donors with HTLV-1 had no evidence of a previous negative result and were identified following the introduction of individual sample testing in 2013. Interestingly, they both had tested negative in pooled screening, but their archive samples were found to be seropositive in retrospective individual testing confirming their infection at the time of the previous donations. Thus, there were 9 incident HTLV infection identified over 20 years of testing. Where the probable source of infection was reported, 58% (138/237) HTLV infections were associated with endemic countries (including the Caribbean region, West Africa, Iran, India, and Japan) and were likely acquired through breast feeding or heterosexual intercourse. In 6 blood donors, epidemiological investigation concluded that infection had probably occurred through self-flagellation (first 3 cases are described here; [18]). Furthermore, all 9 donors with incident infections were born or had lived in the United Kingdom since childhood, and most likely acquired their infection via heterosexual partner in the United Kingdom.

## DISCUSSION

HTLV has been recently recognised as a neglected infection of global concern [6] where HTLV testing will play a vital role in reaching the strategic targets set by the World Health Organization (WHO) [19]. For these reasons, it is important to consider what can be learnt from the experience acquired via the universal blood donation screening for anti-HTLV antibodies introduced in the United Kingdom over 20 years ago [20]. Donations were initially screened in pools (2002–2012) or individually (2013–2016), whereas a change from universal screening to selective testing of donations from first-time donors and without leucodepletion was implemented in 2017. During the first 20-years of screening, over 30 million blood donations have been successfully tested with the Abbott Murex or Prism HTLV-1/HTLV-2 chemiluminescent immunoassay which has demonstrated remarkable overall assay specificity of 99.985%. This is comparable, if not better, than previously evaluated assays used for serological screening of blood donations [21] and exceeds the general recommendations applied for antenatal screening [22].

We have compared data acquired with the different strategies used for HTLV screening of blood donations at presidential scale. Firstly, the excellent specificity of 99.952% based on 6 908 058 donations individually tested and its further increase to 99.999% with the pooled screening of 22 511 003 donations is noteworthy. This represents over a 50-fold reduction in non-specific reactivity with pooling compared with individual sample testing; an issue not previously widely discussed. These data allay concerns that screening results in large numbers of samples with non-specific reactivity thus placing an excessive demand on laboratory services to provide the confirmatory tests. Second, the benefit of the reduced demand for confirmatory testing with the pooled testing strategy, easily outweighs the complexity of pooling at least in low HTLV endemicity areas such as the United Kingdom. Over the 20-year study period, only 5032 repeat reactive donations required confirmatory HTLV testing with 278 (5.5%) confirmed positive. However, <10% of these repeat reactive samples originated from pooled screening (n = 413) and the proportion of these confirmed as HTLV-positive was 27-fold higher with the pooled testing compared to individual screening (47.7% 197/413% vs 1.8%; 81/4619).

Although the confirmatory tests are important to determine true HTLV infection, categorizing signal to cut-off (S/CO) ratios obtained from immunoassays can be used to quickly assess the likelihood of true HTLV-1 infection. Although we were not able to review the S/CO ratios for our confirmed HTLV-1 positive or negative blood donors, a previous study undertaken by a national HTLV reference laboratory with Abbott Architect HTLV assay showed that when the S/CO ratio was below 4.0 the likelihood that HTLV infection will be confirmed, in the United Kingdom, is low, whereas a ratio higher than 20 is highly predictive of true infection [15]. These data have since been confirmed by another UK study [23], the aim being to reduce the number of non-specific reactive samples that required confirmatory testing and to utilise organs from such donors faster for transplantion providing the appropriate risk assessment has been completed. Furthermore, alternative and simplified strategies for confirmatory testing for blood donors have also been recently suggested [24].

The value of pooled testing, and its potential effect on assay sensitivity has been debated for several years. It has not been previously recommended for serological screening of blood donations, whereas it is a generally accepted strategy for molecular-based screening of blood donations [25, 26] and was also successfully pursued to increase testing capacity for severe acute respiratory syndrome coronavirus 2 (SARS-CoV-2(RNA in the early stages of coronavirus disease 2019 (COVID-19) pandemic [27]. Pooling of samples for testing has been recognised as a cost-saving measure, but any cost savings have to be balanced against the risk of failing to detect a positive donation due to sensitivity being compromised in diluted sample [26]. Although not systemically investigated in this study, we are aware of 2 blood donors whose HTLV-infection was noted only after the individual screening was implemented. Further arguments against pooling of samples for serology testing include the potential risk of sample handling errors during the pooling, or when resolving individual samples in each pool, and time taken to complete testing. However, these issues are resolvable via automation as shown with molecular screening of blood donors still applied widely [28]. The counter argument of better specificity seen with pooling has not been previously discussed.

Blood donations screening for 20 years has identified a total of 278 HTLV-infected individuals in England. The highest rates of confirmed cases were obtained when screening first-time and non-leucodepleted component donors; this strategy with a focus on testing first-time donors known to carry the highest risk of transfusion-transmitted infections was shown to be the most cost-effective option in the United Kingdom without compromising blood safety [16]. Furthermore, most HTLV infections were identified in the UK-born individuals who had most likely acquired their infection unknowingly through breast feeding or heterosexual intercourse with an individual associated with an HTLV-endemic country. This is consistent with the results from the first blood donor screening study performed in England in 1991, which identified 4 HTLV-1 seropositive blood donors, all of whom were White, UK-born, females with sexual contact identified as the only risk factor for infection [29]. Similarly, data from an antenatal pilot also showed that whilst high-risk HTLV populations could be identified—for example Afro-Caribbean mothers living in the United Kingdom—a targeted screening approach would likely identify only around 50% of HTLV-1 infected mothers, even if clinicians apply specialist knowledge of risk factors for HTLV infection [12]. Common for all studies, HTLV-infected individuals identified via the screening programmes were asymptomatic, often belonging to ethnic minority groups and unaware of their HTLV status, allowing ongoing viral transmission. Although it is important not to miss the role of HTLV in patients presenting with the most severe disease outcomes such as ATLL and myelopathy, the generally asymptomatic nature of HTLV infection means that diagnosis will most likely need to be based on the epidemiological risk only. The lack of awareness about HTLV infection by the general population and specifically about self-carrier status are major challenges. These underline the importance of universal screening programs with appropriate confirmatory testing, availability of information on HTLV infection to those affected including their families, and further linkage to care pathways [23].

Blood donation screening alone for HTLV will not be sufficient to address the WHO strategic targets aiming to prevent sexual, parenteral and the mother-to-child transmission of HTLV [19]. During our 20-years of HTLV screening of blood donations we have encountered some HTLV-infected women, where potentially the opportunity to prevent the transmission of HTLV to their children was missed. Furthermore, current UK policy recommends screening umbilical cord blood donors for HTLV at the time of collection which delays the HTLV diagnoses of mothers to newborn babies by several weeks; this has resulted in 8 referrals for HTLV-1 infection where the opportunity to prevent vertical transmission of HTLV has been missed. The spread of HTLV-1 infection from mother-to-child can be prevented by advising not to breastfeed, but only if those at risk are identified by health-care workers. The importance of using every opportunity to prevent mother-to-child transmission of HTLV infection should be used, as the risk of infection being more severe is higher if the infection is acquired at young age.

This study focuses on comparing the specificity of HTLV screening assays between pooled and individual testing. Over 30 million blood donations were screened between 2002 and 2021, with a total of 282 confirmed HTLV-positive samples. These data demonstrate the high specificity of HTLV screening assays available since 2002 allaying fears of low specificity which was reported for historical assays [30], still inappropriately cited. Specificity for pooled testing was higher, at 99.999%, compared to 99.985% for individual testing. The non-specific reactivity rate was significantly lower in pooled compared to individual screening (1/100 000 vs 50/100 000). The confirmatory rate consequently increased from 0.9% (30/3349) with individual testing of all blood donations in a low-prevalence setting to 47.7% (197/413) with pooled screening. Despite the higher specificity of pooled testing, individual screening improved detection sensitivity, identifying two HTLV-infected donors missed by pooling. In the presence of leucodepletion, however, these would not have posed a blood safety risk.

Findings confirm that pooled testing is advantageous in low-prevalence settings due to its high specificity and reduced non-specific reactivity, making it more cost-effective. Whether pooling could also be used in resource-poor, high-prevalence countries, requires further investigations.

## Note

***Financial support.*** H.H. acknowledges funding from the National Institute for Health and Care Research (NIHR), grant number NIHR203338 and G.T. the Imperial NIHR Biomedical Research Centre.
